# Bazam: a rapid method for read extraction and realignment of high-throughput sequencing data

**DOI:** 10.1186/s13059-019-1688-1

**Published:** 2019-04-18

**Authors:** Simon P. Sadedin, Alicia Oshlack

**Affiliations:** 1 Bioinformatics, Murdoch Children’s Research Institute, Royal Children’s Hospital, Flemington Road, Parkville, Victoria 3052 Australia; 2 Victorian Clinical Genetics Services, Royal Children’s Hospital, Flemington Road, Parkville, Victoria 3052 Australia; 3 Department of BioScience, University of Melbourne, Parkville, 3050 Australia

## Abstract

The vast quantities of short-read sequencing data being generated are often exchanged and stored as aligned reads. However, aligned data becomes outdated as new reference genomes and alignment methods become available. Here we describe Bazam, a tool that efficiently extracts the original paired FASTQ from alignment files (BAM or CRAM format) in a format that directly allows efficient realignment. Bazam facilitates up to a 90% reduction in the time for realignment compared to standard methods. Bazam can support selective extraction of read pairs from focused genomic regions for applications such as targeted region analyses, quality control, structural variant calling, and alignment comparisons.

**Electronic supplementary material:**

The online version of this article (10.1186/s13059-019-1688-1) contains supplementary material, which is available to authorized users.

## Background

The wide-scale adoption of high-throughput genomic sequencing instruments over the last 10 years has generated vast quantities of genomic data with enormous potential for future use. Genomic data is often stored and exchanged as aligned reads in a coordinate-sorted BAM or CRAM format. This format is common because many applications (such as viewing the alignment or routine variant calling) can utilize it directly. Storage in aligned form, however, has the significant disadvantage that the data is tied to the reference genome and alignment method used. Many results are highly sensitive to these parameters, and combined data sets typically cannot be analyzed together at all unless these parameters are identical. Consequently, to make optimal use of data, users often need to realign the data to a recent genome build and reference. This is resulting in a widespread and growing need for the capability to efficiently realign genomic data.

Realignment of paired reads from aligned data is however both computationally expensive and inconvenient using standard methods. The challenges arise because aligners must access both reads of a pair simultaneously in order to optimally align them. While both reads are usually stored in an alignment file, in a coordinate-sorted file a significant fraction may be distant from each other. In these cases, an expensive random lookup is necessary to read the mate information so that both reads of the pair can be written to the output together. Consequently, the standard practice for realignment involves first extracting all the reads, and then sorting them by read name on disk prior to realignment. While this makes extraction feasible, the process is lengthy and requires substantial resources due to the intermediate steps. Interestingly, Picard Tools [1] offers an alternative method to extract read pairs, in the form of SamToFastq, which avoids the need for these intermediate steps in extracting read pairs. However, this method is not widely used in the community. This is likely because SamToFastq is poorly optimized for memory use, making it impractical for use with large data sets. Additionally, Picard Tools cannot target a specific locus and can only emit a single output stream, causing the process to be bottlenecked by the maximum throughput of a single downstream process (such as alignment). Biobambam [2] is another tool that addresses a similar purpose. Biobambam uses a specialized algorithm and data structure to reduce its memory requirements and increase efficiency, but ultimately relies on storing reads on disk when the number of pending reads to be paired exceeds a threshold. Biobambam also does not offer advanced features such as fully flexible read filtering and is limited to output through a single instance of the aligner.

**Fig. 1:**
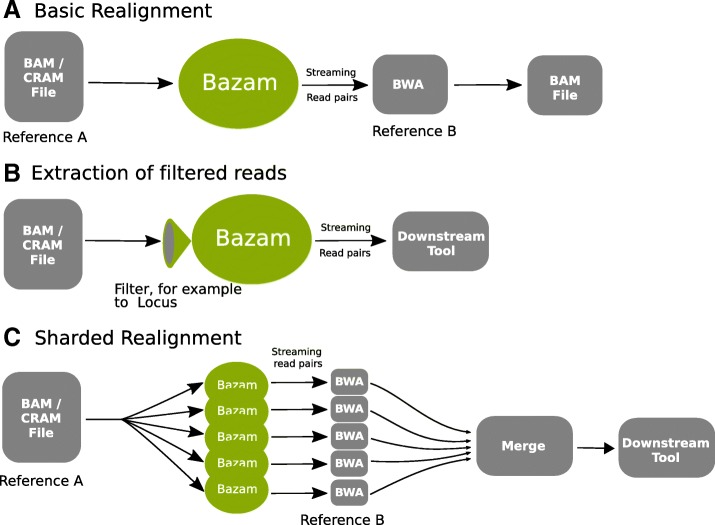
Different configurations for using Bazam. **a** Simple realignment from one reference genome to another without intermediate storage or steps. **b** Extraction of filtered reads such as those overlapping a specific locus. Reads can be streamed to downstream tools directly, or stored in FASTQ format for further processing. **c** Sharded realignment allows for many copies of the aligner to run on different subsets of the data, greatly speeding up realignment

Here we introduce Bazam, an alternative to SamToFastq that optimizes memory use, while offering increased parallelism and other additional features. Bazam increases parallelism by splitting the output streams into multiple paths for separate realignment (Fig. 1). Using this technique, a single-source alignment can be realigned using an unlimited number of parallel aligners, significantly accelerating the process when a computational cluster or cloud computing resource is available.

While realignment is a key application, Bazam also offers utility for any other application relying on detailed read pair information. Example applications include quality control and structural variant calling. Bazam offers two additional features of particular interest: read position tagging and localized extraction. Read position tagging renames reads as they are streamed to include the original alignment position in the name of each read. This feature allows ready comparison between new and old alignment positions after realignment. Localized extraction allows realignment to be limited to reads overlapping specified genomic coordinates. Like realignment, this can be achieved using standard tools. However, these tools do not emit both reads of a pair if only one overlaps a region of interest, and are therefore unsuitable for applications that require both of the reads.

Here we describe the implementation of Bazam and demonstrate that it increases efficiency without compromising accuracy.

## Results

### Bazam design

#### Pairing of reads

The primary challenge in extracting paired reads from BAM and CRAM files arises from the predominant choice of coordinate-sorted ordering for their storage. This format is used because it places all the reads aligned to a given genomic locus in close physical proximity within the file, maximizing efficiency for any analysis focused on short-range variation (such as SNV and indel calling, or visualization in genome browsers). However, coordinate ordering is highly suboptimal for realignment, because a small but significant fraction of reads are located to a large genomic distance from their mate. Consequently, a simple linear scan cannot readily extract both a read and its mate in many cases. One possibility is to retrieve each mate as needed using a random seek within the file to the location of its mate. This strategy is highly inefficient, however, because reads are stored within BAM and CRAM files in a block format such that extracting a single read requires decoding some or all of the other reads from the same block. In practice, such a random seek strategy slows down read extraction by several orders of magnitude.

Bazam retains the efficient linear scan of standard methods. However, instead of performing random lookup of each mate, Bazam stores each read in memory until the mate is encountered naturally. For the majority of pairs, both reads derive from the same biological fragment, which is typically closely matched to the reference genome and therefore a short distance on the genome. In these cases, the mate for a read is encountered soon after the read itself, so that the first read needs to be only briefly stored in memory. Reads aligned at a greater distance from their mate must be buffered for significantly longer. Consequently, Bazam requires enough memory to run such that it can store these reads until their mates are encountered by the linear scan. To reduce the memory load, Bazam does not store the full read data structure in memory when the desired output is in FASTQ format. Instead, Bazam stores only data essential to the FASTQ output. Bazam additionally encodes the in-memory reads to compress the data and reduce memory load.

The worst-case scenario is represented by a small proportion of reads where the mate aligns to a different reference contig (or chromosome). These reads may represent real structural variation within the sample, but can also be generated artefactually in the preparation of sequencing libraries. In these cases, the mate does not resolve at all until its chromosome is encountered by the linear scan. Accordingly, Bazam requires enough memory to store this small proportion of reads for the full duration of the extraction.

By buffering reads, Bazam trades memory for speed. The peak memory required depends on the coverage depth of the alignment, the typical span between paired reads (the insert size distribution) and also on the number of reads whose mates align to different contigs. We observe on typical human whole genome data sequenced at 30× mean coverage depth, that Bazam requires approximately 16–32 GB of RAM. For cancer genomes or other scenarios with many genomic rearrangements, this could potentially increase. However, for many common scenarios, the memory requirement of Bazam remains well within the limitations of the resources available in most modern computing systems.

#### Parallelism and sharding

As computational performance is one of the main goals of Bazam, it is designed with a high level of parallelism internally so that system input/output (I/O) is never blocked. This is achieved by using separate threads for reading the input alignment file, writing the output, and a pool of threads to index and buffer each read so that it can be paired with its mate. To further ensure that performance of Bazam can be scaled, read pairs can be split into multiple streams, which is referred to as “sharding.” In sharded mode, several copies of Bazam are run, with each copy emitting a different subset of the reads. Bazam utilizes the unique read name assigned to each read pair to ensure that the output streams receive mutually exclusive subsets. Specifically, the name of each read is used to generate a hash code and the modulus of this hash code with the total number of shards is used to decide whether a read is processed by a given Bazam instance. Many copies of Bazam can then run on the same alignment file simultaneously, with each one outputting a unique read subset. This arrangement both reduces the peak memory load and increases parallelism, as each shard can be streamed into different instance of the aligner. With a computational cluster or cloud computing facilities, almost unlimited parallelism is achievable with this method. Sharding can also be utilized to downsample data to lower coverage, by omitting one or more output streams from alignment.

#### Output shuffling

In our testing of Bazam, we observed that, on some data sets, use of Bazam could cause significant inflation in memory usage of the downstream aligner software, BWA. This issue was also observed using other tools such as Biobambam. Investigation showed that the memory inflation was linked to the order of the reads and that randomizing the order eliminated the memory inflation. Bazam therefore implements a feature to shuffle extracted reads before writing them to the output. This requires a buffer of reads to be held in memory, increasing Bazam’s memory requirement. However, we find that in some data sets, the increase in usage by Bazam is more than offset by a greater decrease in memory usage by BWA. This effect was not observed in other aligner software that we tested, such as Bowtie2 [3].

#### Memory usage

To reduce memory use, Bazam compresses reads while they are stored for pairing or shuffling. Bazam includes several optional compression methods. The default mechanism uses a 4-bit encoding for bases and the compression library Snappy [4] to encode quality scores. Although 2-bit encoding was considered for bases (allowing 4 possible values), the need to encode possible *N* bases in the reads required at least a 3-bit encoding to store the 5 values. To align these with 8-bit byte boundaries, 2 bases are stored per byte. A future extension could improve this scheme by using 3 bits per base, thereby storing 4 bases in 12 bytes. In our testing, Snappy compression reduces the memory needed to store quality scores to approximately 60% of the original size. Therefore, the overall compression is approximately 55% of the raw size. As a further memory optimization, Bazam scans the BAM index to determine the number of reads aligned to each reference contig. If the mate for a read is aligned to a contig for which no reads are present in the index, Bazam avoids buffering the read in memory. While such reads should not theoretically be present in well-formed BAM files, in practice we have found that BAM files are often filtered using mechanisms that leave such reads in place, resulting in a high memory burden if stored in memory for the duration of the processing.

### Efficiency of realignment on whole genome sequencing

To test the efficiency of Bazam, we applied it to a public whole genome data set (NA12878, 30× mean coverage) released as part of the Genome in a Bottle project [5]. First, we realigned the data set from GRCh37 to GRCh38 using both Bazam and using the standard approach without Bazam. The standard approach consists of first sorting reads using samtools bamshuf (http://www.htslib.org/), then extracting them using samtools bam2fq, and finally realigning using BWA mem [6] and re-sorting the output BAM file using samtools sort. To avoid directly storing intermediate files, this process was constructed using Unix pipes. However, we note that the intermediate sorting stages still write files, resulting in substantial temporary storage requirements. We refer to this process as Sort-Extract-Realign (SER). In this process, we used 16 cores in total as we observed empirically that on our test systems, relatively little improvement in performance was gained by adding additional cores. Picard SamToFastq was run using 32 GB of RAM and given 16 processor cores. However, in this configuration, it failed to complete as it exceeded the allocated memory early in the process. When increased memory was given, anomalies within the data set caused it to abort the process. To enable its performance to be measured, the problematic line in the Picard source code was removed, and the tool was recompiled, which enabled it to complete the processing. The Bazam process consisted of Bazam directly streaming reads into BWA mem, followed by re-sorting with samtools sort.

When using a single instance of BWA, Bazam took similar overall time but reduced the storage required by 75.9% compared to the Sort-Extract-Realign process. In this case, processing time was limited primarily by the speed of alignment rather than Bazam’s ability to pair reads. When run in sharded mode, however, Bazam was able to split reads between 10 copies of BWA, resulting in a time saving of 91%, while still reducing the storage needed by 63.8% (Table 1).

**Table 1:** Comparison of run time, memory, and storage space between Bazam and alternative processes for realignment. Timings encompass the end to end process starting from read extraction and ending with completion of realigned and sorted BAM files

Tool	Storage used	Memory	Effective Cores	Time
Sort-Extract-Realign	282 GB	20 GB	16	13 h, 15 min
Picard SamToFastq	148 GB	78 GB	16	16 h, 14 min
Biobambam bamtofastq	149 GB	30 GB	16	15 h 30 min
Bazam (no sharding)	68 GB	28 GB	16	14 h, 55 min
Bazam 10-way sharding	102 GB	20 GB	160	1 h, 11 min

The total memory used during the non-sharded Bazam realignment peaked at 28 GB. This memory can be broken down into the following: memory used to store reads while they are being paired, memory used by other parts of Bazam, and memory used by BWA. We observed that the peak total memory used by all the Bazam components was approximately 14 GB, with read storage for pairing accounting for approximately 5.4 GB. Much of the remainder is accounted for by large input and output buffers, and internal queuing of data used to ensure high performance. The components outside of Bazam, including BWA and samtools sort, peaked at 14 GB (50%) of memory.

### Accuracy of realignment

We tested the fidelity of Bazam’s read extraction process by comparing Bazam’s output to the expected output using two different methods. First, we converted all reads from the evaluation data set to FASTQ format using the SER method. Then, we aligned these reads to GRCh37 using BWA mem and re-extracted to FASTQ format using Bazam. Comparison of the two FASTQ data sets found that reads were identical, showing that Bazam reproduces FASTQ with perfect fidelity.

To investigate any unexpected effects resulting from realignment with Bazam, we first realigned the SER-extracted FASTQ to GRCh37, to create an updated alignment using our local alignment configuration. Next, we realigned this updated alignment, with Bazam. These steps ensured that both alignments with and without Bazam used identical reference genomes and aligner settings, so that these factors did not cause artefactual differences.

We then compared the alignments with each other, by applying Bazam’s read position tagging feature. The feature alters read names during realignment to carry the original alignment position. In this way, reads in the new alignment could be readily checked against their old position to identify reads that “moved.”

The comparison between the Bazam and updated realignments revealed a total of 13.7 m (1.7%) reads that changed position after Bazam realignment. We hypothesized based on previous studies [7] that this may be caused by ambiguously positioned reads aligning differently due to altered input order. Consistent with this hypothesis, we identified that of the repositioned reads, 92.8% had mapping quality of 30 or less, suggesting their alignments are subject to significant ambiguity. We investigated the moved reads that had high mapping quality and observed that many of the these were mapped to repeat masker regions (Additional file 1: Table S1) and in many cases were in fact subject to ambiguity despite receiving high mapping quality from BWA. Based on these results, we concluded that Bazam realignment has a minimal effect on reads with unambiguous mapping positions, and while reads with ambiguous positions may be repositioned, this is likely due to known behavior of BWA, rather than Bazam itself.

### Application to repeat expansion calling

As an example of Bazam’s utility for aiding downstream analysis tools such as complex variant calling, we applied Bazam to STRetch [8], a method for detection of short tandem repeat expansions (STRs) in genomic data. The first step in STRetch selects reads aligning to more than 400,000 known STR repeat regions (as well as any unmapped reads) and then realigns these reads to artificial decoy sequences containing short tandem repeats. When run on pre-aligned data, STRetch extracts all reads within 800 bp of each known STR region. This window is chosen to be wide enough to capture both reads of the majority of pairs that fall into the STR region. Nonetheless, some pairs are mapped widely enough apart that they may be missed. We replaced this implementation with Bazam’s local extraction feature and tested the accuracy and efficiency.

When run using the default read extraction method on the same whole genome sample, STRetch took 6 h and 7 min. The unsharded Bazam method reduced the time required to 2 h and 27 min. This improvement is achieved partly by avoiding intermediate FASTQ extraction, but also by eliminating the additional window required for scanning of candidate STR reads. Bazam makes the expanded window unnecessary because it guarantees to output both reads of a pair, even if only one overlaps the extraction window, demonstrating the utility of the localized extraction feature. When run using sharded mode with six copies of BWA, STRetch finished in 1 h and 24 min. STRetch primarily derives its sensitivity from its ability to align reads from STR regions to the decoy sequences. Hence, we compared STRetch performance between Bazam and standard alignment methods by counting the reads that were aligned to each decoy sequence. We found that Bazam was able to align 3.4% more reads to the decoy sequences than the standard alignment process. Therefore, we conclude that alignment using Bazam increases both speed and accuracy in the case of STRetch.

### Wider applications

While we have primarily developed Bazam with realignment in mind, any application where paired reads are needed can benefit. In particular, we note that many algorithms for complex and structural variant calling are highly dependent on read pair information and hence could benefit from building on this method. Quality control statistics derived from read pair information can also be calculated more efficiently using Bazam than standard methods. Finally, the ability to tag read names with previous alignment information is also useful for benchmarking and comparing alignment software.

## Conclusion

Bazam offers a simple, yet effective, tool that enables a significant increase in efficiency and decrease in time required to realign existing genomic data. This has widespread practical utility, as the need to reprocess data onto new genome builds with updated alignment software is becoming increasingly prevalent. Bazam also has many other potential uses for applications where full read pair information is needed, especially where extraction from localized regions of the genome is of interest. Bazam is open source software and is available at https://github.com/ssadedin/bazam.

## Methods

### Software implementation

Bazam is implemented using Groovy, a modern language derived from Java and which shares most properties with Java including platform independence and very high performance. Bazam uses HTSJDK (https://github.com/samtools/htsjdk) for the underlying BAM and CRAM parsing operations. To enable high concurrency, Bazam employs actor-based concurrency based on the GPars framework (http://www.gpars.org/).

Source code for Bazam used in producing the results presented is available at 10.5281/zenodo.2590831 and is freely available under the Lesser General Public License (LGPL). Methods can be found at https://gitlab.com/ssadedin/bazam-paper-methods.

## Additional file

Additional file 1: **Table S1.** Additional details of the methods, data sources and statistics regarding realignment of reads. (DOCX 21 kb)
