# Supplementary material for: Bazam: a rapid method for read extraction and realignment of high-throughput sequencing data
Source: Genome Biol. 2019 Apr 18;20:78. doi: 10.1186/s13059-019-1688-1 (PMC6472072; doi:10.1186/s13059-019-1688-1)
Supplement: Supplementary file 1 — Table S1. Additional details of the methods, data sources and statistics regarding realignment of reads. (DOCX 21 kb) [file 13059_2019_1688_MOESM1_ESM.docx]

Supplementary Material for Bazam : A rapid method for read extraction and realignment of high throughput sequencing data

# Simon P Sadedin^1,2^, Alicia Oshlack^1,3^

^1^Bioinformatics, Murdoch Children’s Research Institute, Royal Children's Hospital, Flemington Road, Parkville, Victoria 3052 Australia

^2^Victorian Clinical Genetics Services, Royal Children's Hospital, Flemington Road, Parkville, Victoria 3052 Australia

^3^Department of BioScience, University of Melbourne, Parkville 3050, Australia

Corresponding authors: SS [simon.sadedin@mcri.edu.au](mailto:simon.sadedin@mcri.edu.au), AO [alicia.oshlack@mcri.edu.au](mailto:alicia.oshlack@mcri.edu.au)

## 1. Evaluation Data

The evaluation data set was downloaded from the NIST “Genome in a Bottle” FTP site at the following location:

ftp://ftp-trace.ncbi.nlm.nih.gov/giab/ftp/data/NA12878/NIST_NA12878_HG001_HiSeq_300x/RMNISTHS_30xdownsample.bam

## 2. Evaluation Scripts / Pipeline

The evaluation was performed using pipelines constructed with the Bpipe framework (<http://bpipe.org>). The scripts used are deposited in the following public Gitlab repository:

https://gitlab.com/ssadedin/bazam-paper-methods

Recording of timings for analyses was accomplished using the Bpipe “stats” feature which reports the time taken by each stage of an analysis pipeline. Timing statistics were captured and are stored in ‘stats.txt’ files in the repository.

The execution environment consisted of a Torque cluster with approximately 500 cores managed by the Murdoch Childrens Research Institute, comprising 16 nodes with each node having 32 – 48 cores and 256GB RAM. Jobs were submitted using Bpipe to the computational cluster, taking care to run each job when the cluster was not overloaded so that there would not be influence from overloading of cluster nodes.

## 3. Reads Repositioned after Bazam Realignment

Reads were first aligned from the Illumina Platinum Genomes raw alignment to GRCh37 using the standard approach (see manuscript) to create an updated alignment. This alignment was then subject to realignment with Bazam using the same reference genome and aligner settings. Table 2 shows the number of reads that aligned to positions that were different in the penultimate and final alignment.

Table S1: Comparison of read positions after realignment using Bazam and BWA with identical alignment settings to the original aligner.

| Category | Number of Moved Reads |
| --- | --- |
| Total Reads | 800,000,000 |
| Reads Moved to different Position in Realignment | 13,710,675 (1.7%) |
|  |  |
| *Of all Moved Reads* |  |
| Multimapping Moved Reads | 10,934,279 (79.8%) |
| Poor Mapping Moved Reads (1 < MAPQ <30) | 1,794,974 (13.1%) |
| Secondary Alignments | 192,007 (1.4%) |
| Mate moved for expected reason | 69,515 (0.51%) |
| Soft Clipping Differences | 54,899 (0.4%) |
| Multiple Mismatches | 42,764 (0.31%) |
|  |  |
|  |  |
| **Total Unexplained Moved Reads** | 215,494 (0.03%) |

## 
